# Tracing *Strongyloides stercoralis* transmission and risk factors from schoolchildren to household in Northwest Ethiopia

**DOI:** 10.1371/journal.pntd.0014857

**Published:** 2026-11-02

**Authors:** Tadesse Hailu, Getaneh Alemu, Anemen Ayehu, Hiwot Tadesse, Zelalem Mehari, Hilina Motbainore, Genet Gedamu, Endalkachew Nibret

**Affiliations:** 1 Department of Medical Parasitology and Entomology, College of Medicine and Health Sciences, Bahir Dar University, Bahir Dar City, Ethiopia; 2 Department of Epidemiology and Biostatistics, School of Public Health, College of Medicine and Health Sciences, Bahir Dar University, Bahir Dar City, Ethiopia; 3 Department of Medical Microbiology, College of Medicine and Health Sciences, Bahir Dar University, Bahir Dar City, Ethiopia; 4 Department of Environmental Health, School of Public Health, College of Medicine and Health Sciences, Bahir Dar University, Bahir Dar City, Ethiopia; 5 Department of Biology, Science College, Bahir Dar University, Bahir Dar City, Ethiopia; Washington University School of Medicine, UNITED STATES OF AMERICA

## Abstract

**Background:**

Strongyloides *stercoralis* is a neglected soil-transmitted helminth with a complex life cycle that enables both autoinfection and environmental transmission. Despite its public health importance, the burden and transmission dynamics of strongyloidiasis remain poorly understood in endemic settings such as Ethiopia, particularly at the household level. Hence, this study aimed to trace the transmission of *S. stercoralis* and associated factors from infected schoolchildren to their households in Northwest Ethiopia.

**Methodology:**

A school-based cross-sectional study was conducted from 2024 to 2025 in Northwest Ethiopia. A total of 273 schoolchildren were randomly selected from three schools and screened for *S. stercoralis*. Children who tested positive were considered index cases, and all consenting household members were subsequently screened. Stool samples were examined using Kato-Katz, the Baermann concentration technique, and agar plate culture. Data on socio-demographic characteristics and potential risk factors were collected using structured questionnaires. Bivariate and multivariable logistic regression analyses were performed to identify factors associated with infection.

**Findings:**

Out of 273 school children screened, the prevalence of *S. stercoralis* was 22.7%. Among **80** household members examined, 71.3**%** were infected with *S. stercoralis*. School children who lived in houses with mud floors (AOR = 2.91; p = 0.019), had close contact with dogs (AOR = 2.54; p = 0.006), lived in areas with clay soil (AOR = 2.98; p = 0.036), participated in irrigation activities (AOR = 3.72; p = 0.008) and wore shoes (AOR = 3.67; p = 0.017) were significantly associated with *S. stercoralis* infection.

**Conclusions:**

This study demonstrates a substantial burden of *S. stercoralis* infection among schoolchildren and an exceptionally high prevalence among their household members. The findings underscore the importance of environmental, behavioral, and zoonotic factors in sustaining transmission. These results emphasize the urgent need for integrated control strategies to include household-level screening and treatment.

## Author summary

This study aimed to investigate the transmission dynamics and risk factors of *Strongyloides stercoralis* infection among schoolchildren and family members of infected children in Northwest Ethiopia. Samples of stool were collected from both groups and examined by standard parasitological diagnostic techniques. Results showed a high infection burden in schoolchildren and a significantly higher prevalence in their household members, providing strong evidence for household clustering of infection. Several risk factors were identified, including living in houses with mud floors, close contact with dogs, playing with soil, and types of soil. Moreover, important behavioral determinants that contributed to exposure included lack of proper frequency of latrine utilization, participation in irrigation activities, and inconsistent shoe wearing. The findings underscore that a combination of environmental contamination and behavioural practices within households and communities contributes to transmission of *S. stercoralis*. The study indicates the need for integrated control strategies, including sanitation improvement, health education, and behavioural change accompanied by community-based deworming interventions targeting children and their family members.

## Introduction

*Strongyloides stercoralis*, the causative agent of strongyloidiasis, is a re-emerging, neglected tropical disease that infects both humans and a wide range of animals, including dogs, cats, cattle, sheep, goats, horses, and pigs [1]. Globally, an estimated 613.9 million people are infected, with the majority of cases concentrated in Southeast Asia, the Western Pacific, and Africa [2]. In Ethiopia, recent reviews report a prevalence ranging from 0.21% to 55.7%, highlighting the disease’s significant public health impact [3].

Humans acquire *S. stercoralis* infection through autoinfection, in which filariform larvae penetrate the intestinal mucosa or perianal skin, or through heteroinfection [4]. Heteroinfection is facilitated by warm, moist soil, poor sanitation, and close contact with animal or human feces containing infective larvae. Schoolchildren are particularly vulnerable due to barefoot walking, frequent soil contact, and close interaction with domestic animals [5].

The clinical spectrum ranges from asymptomatic cases to life-threatening disseminated infections, particularly among immunosuppressed individuals. Filariform larvae can transport gram-negative bacteria through the bloodstream, leading to severe bacterial infections and high fatality rates [6]. Diagnosis remains challenging due to low parasite loads, irregular larval shedding, and the lack of a highly sensitive “gold standard” method, highlighting the need for combining multiple diagnostic approaches to accurately detect infections [7–9].

Strongyloidiasis is preventable through improved hygiene and sanitation, footwear use, safe disposal of feces, treated water access, and minimizing close contact with animals [10]. However, control is hampered by the parasite’s zoonotic potential, environmental persistence, and gaps in identifying infection sources. Domestic animals, particularly dogs, have been implicated as potential reservoirs, with phylogenetic studies supporting possible cross-transmission between humans and dogs [11]. The infective filariform stage can also persist in soil, facilitating environmental transmission [12].

Given its public health importance, the World Health Organization has included *S. stercoralis* in its 2020–2030 roadmap for soil-transmitted helminth control [13]. Untreated infections in children contribute to impaired physical and cognitive development, poor school performance, malnutrition, and perpetuation of poverty [14,15].

Despite the recognized burden of *S. stercoralis* in endemic regions, there is limited evidence on the household-level transmission dynamics in Ethiopia. While schoolchildren are often identified as sentinel hosts, few studies have systematically investigated how infections spread within their families, including the roles of sanitation, hygiene practices, and domestic animal contact. As a result, the transmission dynamic, relatively, the contribution of household members to sustaining *S. stercoralis* infections in the community and at household level remains poorly understood. Addressing this gap is critical for designing effective, targeted interventions to prevent and control strongyloidiasis in endemic communities. Hence, the aim of this study was to trace the transmission of *S. stercoralis* and associated factors from infected schoolchildren to their family members at the household level in Northwest Ethiopia and to generate evidence that can inform targeted prevention and control strategies for strongyloidiasis in endemic communities.

## Methods

### Ethics statement

Ethical approval was obtained from the Ethical Review Board of the College of Medicine and Health Sciences, Bahir Dar University (protocol number 003/2024). Supportive letters were secured from the Amhara National Regional Education Bureau, Bahir Dar City and the Bahir Dar Zuria District Health Offices. All procedures performed in this study involving human participants were in accordance with the ethical standards of the institutional and/or national research committee and with the 2024 Helsinki Declaration. Written informed consent was obtained from all individual participants included in the study or from their legal guardians where applicable. Individuals who tested positive for any intestinal parasitic infection were referred to a physician for appropriate treatment.

### Study design and setting

A school-based cross-sectional study was conducted from 2024 to 2025 in Bahir Dar City and the Bahir Dar Zuria District, Northwest Ethiopia. The study area includes both urban and rural settings with varying sanitation and hygiene conditions, where soil-transmitted helminth infections, including *S. stercoralis,* are endemic.

### Study population and sampling

The study population comprised schoolchildren enrolled in selected primary schools and their household members. Three schools (Kulkual and Woramit primary schools in Bahir Dar City and Robit primary school in the Bahir Dar Zuria district) were selected randomly. All schoolchildren within the 6–14 age range were the target population. The number of study participants was proportionally allocated to each school based on the number of students within the accepted age category. All schoolchildren who provided stool samples and whose parents or guardians gave informed consent were included in the study. Household members of confirmed positive schoolchildren who were present during the study period and consented to participate were also included. Schoolchildren and their families who were taking an anthelminthic drug during the data collection period, were unable to provide stool samples, and/or declined participation were excluded from the study.

The sample size was estimated using a previous Baermann concentration technique (BCT) and agar plate culture (APC)-based *S. stercoralis* prevalence (13.6%) study [9], a 95% confidence interval (CI), a 5% margin of error, and a 1.5 design effect [16].

A total of 297 schoolchildren were randomly selected and screened for *S stercoralis* infection. Children who tested positive for *S. stercoralis* were considered index cases, and all available household members of these index cases were subsequently included and screened to assess household-level transmission. A participant was considered positive for *S. stercoralis* if either the BCT or APC yielded a positive result. Thus, discordant results between the two methods were classified as positive.

### Data collection

Data on socio-demographic characteristics and potential risk factors were collected using a pre-tested structured questionnaire administered to participants or guardians. Variables included age, sex, family size, educational status, sanitation facilities, water source, hygiene practices (such as handwashing and shoe-wearing habits), contact with domestic animals, and involvement in agricultural activities. Stool samples were also collected using a leak-proof stool container.

### Stool sample collection and laboratory analysis

Fresh stool samples were collected from all study participants using clean, labeled containers. All stool samples were examined using three diagnostic methods. To enhance the detection of *S. stercoralis*, each sample was examined using a combination of diagnostic techniques, acknowledging the absence of a single gold standard method. For the specific detection of *S. stercoralis larvae*, the Baermann concentration technique (BCT) and agar plate culture (APC) were used due to their higher sensitivity. The Kato-Katz (KK) technique was employed primarily for the detection of other intestinal helminths.

For the Baermann concentration technique (BCT), approximately 10–15 g of fresh stool sample was weighed and mixed with water and powdered charcoal. The mixture was transferred onto a Petri dish lined with a double layer of paper towels and then covered with a single layer of paper towel. It was incubated at 26°C for 24 hours. After incubation, the stool sample was placed in a sieve and suspended in a funnel containing warm water, which was connected to a rubber tube. The setup was left undisturbed for one hour to allow larvae to migrate into the water. The filtrate was then collected into a tube and centrifuged at 2000 rpm for 5 minutes. Finally, the sediment was mixed, transferred onto a microscope slide, and examined under a microscope using 4 × , 10 × , and 40 × objectives for the detection of *S. stercoralis* larvae [9].

For the agar plate culture (APC), approximately 3 g of fresh stool sample was placed at the center of an agar plate (prepared from beef extract, peptone, sodium chloride, agar powder, and distilled water) in a Petri dish. The plate was sealed with adhesive tape and incubated at 26°C for 48 hours. The surface of the agar plate was examined daily using a dissecting microscope or by the naked eye for the presence of characteristic furrows or tracks produced by migrating larvae [17]. After incubation, the adhesive tape was carefully removed, and 5 mL of 10% formalin was added to the surface of the agar plate. The plate was left for 5 minutes to allow fixation of larvae. The formalin suspension was then collected into a test tube and centrifuged at 1500 rpm for 5 minutes. Finally, the sediment was examined under a microscope for the detection of *S. stercoralis* larvae, which were differentiated from hookworm larvae using standard morphological criteria and a diagnostic chart [18].

In the Kato–Katz (KK) technique, a stool sample was first pressed through a mesh screen to remove large particles. Approximately 41.7 mg of sieved stool was then transferred into a template placed on a microscope slide, ensuring that the template hole was completely filled. The template was carefully removed, and the stool sample was covered with a cellophane strip previously soaked in a glycerol–malachite green solution. The preparation was gently pressed using another slide to evenly spread the sample. The KK thick smears were examined for soil-transmitted helminths (STHs) eggs, and infection intensity was quantified based on eggs per gram (EPG) of stool. The intensity of *soil* was classified as light, moderate, and heavy according to World Health Organization (WHO) criteria [19].

### Data quality assurance

Data collectors and laboratory personnel were trained prior to data collection. Standard operating procedures were strictly followed during stool collection, processing, and examination. A subset of samples was re-examined by experienced laboratory professionals to ensure consistency and reliability. Completed questionnaires were checked daily for completeness and accuracy. To maintain the reliability of the study findings, 10% of the KK slides were randomly selected and reexamined by a third laboratory personnel member who was blind for the first stool examination. The principal investigator also checked the discordant results and wrote the final result.

### Data management and statistical analysis

Data was entered, cleaned using Epi Info version 7, and analyzed by Statistical Package for Social Sciences (SPSS) version 26. Descriptive statistics were used to summarize the prevalence of *S. stercoralis* infection among schoolchildren and household members. Bivariate and multivariable logistic regression analyses were performed to identify factors associated with infection. Variables with a p-value <0.05 were considered statistically significant, and adjusted odds ratios (AORs) with 95% confidence intervals (CIs) were reported.

## Results

Out of 297 schoolchildren initially recruited, 273 were included in the final analysis, yielding a response rate of 91.9%. The non-response rate was 8.1%, primarily due to participants being unable to provide enough stool samples. In the present study, 273 (139 females and 134 males) study participants were recruited. Similarly, urban residents (65.9%) were more represented compared to rural dwellers (34.1%). The majority of the study participants (46.1%) were in the age range 9–11 years (Table 1). Following *S. stercoralis*-positive schoolchildren, 80 family members were recruited in 23 households. All participants were recruited in the rural settings. The majority of the families (73.8%) were above 14 years of age. Male participants of the family members accounted for 58.8% (Table 1).

**Table 1:** Socio-demographic characteristics schoolchildren and their families in Northcentral Ethiopia (n = 353).

Variables	Category	Frequency	
		N	%
Schoolchildren			
Age in years	6-8	47	17.2
	9-11	126	46.1
	12-14	100	36.6
Sex	Male	134	49.1
	Female	139	50.9
Residence	Rural	93	34.1
	Urban	180	65.9
Primary school	Kulkual	88	32.2
	Robit	77	28.2
	Woramit	92	33.7
	Yinesa	13	5.9
**Schoolchildren total**		**273**	**100.0**
Their families			
Age in years	<6	9	11.2
	6-14	12	15.0
	>14	59	73.8
Sex	Female	33	41.2
	Male	47	58.8
Residence	Rural	80	100.0
**Family total**		**80**	**100.0**
**Grand total (schoolchildren + families)**		**353**	**100.0**

### Prevalence of intestinal helminths

Intestinal parasites were detected in 273 schoolchildren and 80 families of *S. stercoralis*-positive schoolchildren. About 32.9% (116/353) of *S. stercoralis* positive cases were identified by both BCT and APC. From the total 116 positives, BCT and APC detected 67.2% (78/116) and 60.3% (70/116) of cases, respectively, highlighting the superior sensitivity of these techniques for detecting *S. stercoralis* (Table 2).

**Table 2:** Prevalence of intestinal helminthic infections among schoolchildren and their family members across different diagnostic methods in Northwest Ethiopia (n = 353).

Types of parasites	Types of diagnostic methods			
	KK	BCT	APC	Total
*S. stercoralis*	–	70 (19.8)	78 (22.1)	116 (32.9)
Hookworm *spp.*	62 (17.6)	–	85 (24.1)	94 (26.6)
*S. mansoni*	35 (9.9)	–	–	35 (9.9)
*lumbricoides*	38 (10.8)	–	–	38 (10.8)
*T. trichiura*	2 (0.6)	–	–	2 (0.6)
H. nana	4 (1.1)	–	–	4 (1.1)
Fascial *spp.*	2 (0.6)	–	–	2 (0.6)

*KK = Kato-Katz, BCT = Baermann concentration technique, APC = Agar plate culture

*Hookworm* spp. were the second most prevalent parasites, detected in 26.6% (94/353) of the study participants. Detection varied by method, with 17.6% (62/353) identified using KK and a higher proportion, 24.1% (85/353), detected by APC, indicating improved recovery with culture-based methods.

Other helminths were primarily detected using the KK technique. *S. mansoni* had a prevalence of 9.9% (35/353), while *A. lumbricoides* was detected in 10.8% (38/353) of participants. Less prevalent infections included *T. trichiura* at 0.6% (2/353), *H. nana* at 1.1% (4/353), and *Fasciola spp*. at 0.6% (2/353) (Table 2).

### Prevalence of *S. stercoralis* among schoolchildren

The overall prevalence of *S. stercoralis* among schoolchildren, using the combined results of the Baermann concentration technique (BCT) and agar plate culture (APC), was 22.7% (62/273). Among these, 11 children were positive by both methods, demonstrating partial overlap but also complementary detection capacity of the two techniques. Overall, 15.8% (43/273) of the children were positive by BCT, while 11.0% (30/273) were positive by APC.

When stratified by sex, males showed a higher prevalence than females. Among males (n = 134), 17.9% were positive by BCT and 12.7% by APC, with an overall prevalence of 25.4%. In contrast, females (n = 139) had lower positivity rates, with 13.7% by BCT and 9.4% by APC, resulting in an overall prevalence of 20.9%.

Analysis by age group revealed that children aged 9–11 years had the highest overall prevalence (26.2%), followed closely by those aged 6–8 years (25.5%). The lowest prevalence was observed in the 12–14 years age group (17%). Similarly, BCT detected the highest proportion in the 9–11-year group (19.1%), while APC detection was also relatively higher in younger age groups.

Regarding residence, children from rural areas exhibited a higher overall prevalence (26.9%) compared to their urban counterparts (20.6%). Interestingly, APC detected a higher proportion of infections in rural children (20.4%) than in urban children (6.1%), whereas BCT showed relatively comparable detection between rural (15.1%) and urban (16.1%) participants (Table 3).

**Table 3:** Prevalence of *S. stercoralis* among schoolchildren across their demographic factors in Northwest Ethiopia (n = 273).

Types of parasites	Category	N	*S. stercoralis* positivity (N, %)		
			BCT	APC	Total
**Sex**	**M**	134	24 (17.9)	17 (12.7)	34 (25.4)
	**F**	139	19 (13.7)	13 (9.4)	28 (20.9)
**Age in years**	**6-8**	47	8 (17.0)	7 (14.9)	12 (25.5)
	**9-11**	126	24 (19.1)	14 (11.1)	33 (26.2)
	**12-14**	100	11 (11)	9 (9)	17 (17)
**Residence**	**Rural**	93	14 (15.1)	19 (20.4)	25 (26.9)
	**Urban**	180	29 (16.1)	11 (6.1)	37 (20.6)
**Total**		**273**	**43 (15.8)**	**30 (11.0)**	**62 (22.7)**

*BCT = Baermann concentration technique, APC = Agar plate culture

### Prevalence of *S. stercoralis* among family members

Among the 62 schoolchildren found positive for *S. stercoralis*, 23 households agreed to participate, providing stool samples from 80 family members. Of these, 57 individuals (71.3%) were infected when results from both diagnostic methods were considered together. Eighteen family members were positive by both Baermann concentration technique (BCT) and agar plate culture (APC), showing that while there is some overlap, each method also identifies additional cases. In general, APC detected more infections (60.0%) than BCT (33.8%).

When results were examined by sex, males showed 23.4% positivity by BCT and 66.0% by APC, giving an overall prevalence of 70.2%. Among females, 48.5% were positive by BCT and 51.5% by APC, with a slightly higher combined prevalence of 72.7%.

Considering age groups, infection was observed across all categories. The highest prevalence was recorded among individuals aged 6–14 years (75.0%), where both BCT and APC detected equal proportions (50.0%). Those older than 14 years also had a high prevalence (72.9%), with APC identifying more cases (62.7%) compared to BCT (33.9%). Children under 6 years had the lowest prevalence (55.6%); however, APC still detected a considerable proportion of infections (55.6%), whereas BCT identified only 11.1% (Table 4).

**Table 4:** Prevalence of *S. stercoralis* among family members across their demographic factors in Northwest Ethiopia (n = 80).

Types of parasites	Category	N	*S. stercoralis* positivity (N, %)		
			BCT	APC	Total
**Sex**	**M**	47	11 (23.4)	31 (66.0)	33 (70.2)
	**F**	33	16 (48.5)	17 (51.5)	24 (72.7)
**Age in years**	<6	9	1 (11.1)	5 (55.6)	5 (55.6)
	6-14	12	6 (50.0)	6 (50.0)	9 (75.0)
	>14	59	20 (33.9)	37 (62.7)	43 (72.9)
**Total**		**80**	**27 (33.8)**	**48 (60.0)**	**57 (71.3)**

*BCT = Baermann concentration technique, APC = Agar plate culture

### Factors associated with *S. stercoralis*

In the multivariate analysis, participants living in houses with mud floors were more likely to be infected compared to those living in houses with cement floors (AOR = 2.91; 95% CI: 1.19–7.13; p = 0.019). In the same way, individuals who had close contact with dogs had higher odds of infection than those who did not (AOR = 2.54; 95% CI: 1.30–4.95; p = 0.006).

Environmental exposure also showed a significant effect. Those living in areas with clay soil had higher odds of infection compared to those living in areas with black soil (AOR = 2.98; 95% CI: 1.07–8.30; p = 0.036). Participation in irrigation activities was another important factor, as individuals involved in irrigation were more likely to be infected (AOR = 3.72; 95% CI: 1.40–9.88; p = 0.008).

In terms of personal behavior, participants who only sometimes wore shoes had higher odds of infection compared to those who always wore shoes (AOR = 3.67; 95% CI: 1.26–10.70; p = 0.017).

On the other hand, playing with soil (p = 0.195) and frequency of latrine utilization (p = 0.816) did not show a statistically significant association with infection in this study (Table 5).

**Table 5:** Univariate and multivariate analysis of factors associated with *S. stercoralis* infection among schoolchildren across their demographic factors in Northwest Ethiopia (n = 273).

Variables	Categories	*S. stercoralis* infection [N (%)]		COR (95%CI)	*P-* *Value*	AOR (95%CI)	*P- value*
		Yes	No				
**Status of house floor**	**Mud**	51	124	3.62 (1.74-7.52)	0.001	2.91 (1.19-7.13	0.019*
	**Cement**	10	88	1		1	
**Having close contact with dogs**	**Yes**	29	61	2.24 (1.25-4.02)	0.007	2.54 (1.30-4.95)	0.006*
	**No**	32	151	1		1	
**Playing with soil**	**Yes**	5	57	4.12 (1.57-10.80)	0.004	2.12 (0.68-6.58)	0.195
	**No**	56	155	1		1	
**Soil type**	**Clay soil**	54	132	4.68 (2.03-10.78	<0.001	2.98 (1.07-8.3)	0.036*
	**Black soil**	7	80	1		1	
**Frequency of Latrine utilization**	**Sometimes**	44	103	2.74 (1.47-5.10)	0.001	1.13 (0.40-3.20)	0.816
	**Always**	17	109	1		1	
**Participate in irrigation**	**Yes**	55	148	3.96 (1.62-9.67)	0.002	3.72 (1.40-9.88)	0.008*
	**No**	6	64	1		1	
**Frequency of shoes wearing**	**Sometimes**	47	109	3.17 (1.65-6.12	0.001	3.67 (1.26-10.70)	0.017*
	**Always**	14	103	1		1	

## Discussion

This study revealed a high burden of *S. stercoralis* infection among schoolchildren and their family members in Northwest Ethiopia, highlighting its ongoing public health importance. In the present study, the overall prevalence of *S. stercoralis* among schoolchildren was 22.7%, which was consistent with a previous report of 20.7% in the Amhara Region [20]. This report is also higher than earlier reports, 15.8% in Malaysia [21] and 17.4% in Rwanda [22]. However, the present finding is lower than the previous reports of 39.0% in northwest Ethiopia [23] and 48.6% in the rural community of Ethiopia [8]. The difference could be attributed to variations in study settings, diagnostic methods, population characteristics, and environmental conditions. Studies reporting higher prevalence, such as those conducted in northwest Ethiopia and rural communities, often employed more sensitive diagnostic techniques like BCT, APC, and polymerase chain reaction (PCR), which increase detection rates compared to a combination of BCT and APC only. In addition, rural settings with poor sanitation, limited access to clean water, and frequent contact with contaminated soil may facilitate higher transmission. On the other hand, the relatively lower prevalence observed in the present study and in countries like Malaysia and Rwanda may reflect differences in climate, socioeconomic status, hygiene practices, and the implementation of deworming and public health interventions. Variations in sample size, age distribution of participants, and study period may also contribute to the observed discrepancies across studies.

Notably, the prevalence among schoolchildren (22.7%) and especially among their family members (71.3%) underscores the potential for intense household-level transmission. This finding strongly supports the concept of intra-household clustering and continuous transmission cycles within endemic communities. Similar patterns have been reported in previous studies, where *S. stercoralis* infection showed significant clustering within households due to shared environmental exposure and common behavioral practices. For instance, individuals living in the same household had a higher likelihood of infection, indicating localized transmission dynamics [24]. Likewise, molecular evidence of overlapping *S. stercoralis* populations among humans and domestic animals within the same households, further supporting household-level transmission [25]. In addition, close contact, poor sanitation, and shared contaminated environments contribute to persistent reinfection cycles within families [26]. Together, these findings reinforce that households serve as key transmission units, sustaining infection reservoirs in endemic settings.

The markedly higher detection of *S. stercoralis* using BCT and APC, compared to the complete absence of detection by Kato–Katz, confirms the well-documented low sensitivity of routine microscopy for this parasite. In the present study, APC identified more cases than BCT, particularly among family members, indicating its superior sensitivity in detecting low-intensity or chronic infections. The complementary nature of these methods further emphasizes the need for combined diagnostic approaches in epidemiological studies and control programs [7–9].

Demographic variations showed that infection was slightly higher among males than females. This report is consistent with a previous report [27–29]. The observed pattern can be justified by increased outdoor exposure among boys and school-aged children, including frequent barefoot walking, playing in contaminated soil, and lower adherence to personal hygiene practices, all of which enhance the risk of contact with infective larvae. In addition, children in this age group are more likely to engage in activities such as farming assistance or playing in moist environments that favor parasite survival.

The higher *S. stercoralis* prevalence (26.9%) observed among rural residents in the present study, consistent with the previously reported 41.1% in northwest Ethiopia [23], can be explained by environmental and socioeconomic factors. Rural communities often face inadequate sanitation facilities, reliance on open defecation, limited access to safe water, and closer contact with contaminated soil and domestic animals. These conditions create a favorable environment for the maintenance and transmission of *S. stercoralis*, thereby increasing the risk of infection compared to urban settings.

The overall prevalence (32.9%) among children and their family members is lower than the previously reported 55.7% in a rural community of Ethiopia [8]. This discrepancy may reflect differences in diagnostic approaches and environmental exposure. Studies that report higher prevalence often utilize more sensitive techniques such as molecular techniques, which enhance the detection of *S. stercoralis* compared to APC and BCT [7,9]. In addition, differences in study population structure, sample size, and seasonal timing of data collection may also contribute to the observed variation in prevalence between studies. The extremely high prevalence (71.3%) of *S. stercoralis* infection among family members suggests active transmission within households. This could occur through shared environmental exposure, poor hygiene practices, and possible zoonotic transmission.

In the present study, environmental and behavioral factors were important determinants of infection. For instance, living in houses with mud floors, residing in areas with clay soil, and participating in irrigation activities significantly increased the odds of infection. These conditions likely favor larval survival and enhance human–soil contact. This report was consistent with a previous report in [5]. In addition, inconsistent use of footwear was strongly associated with infection, reflecting the importance of skin penetration as the primary route of transmission. This finding was consistent with an earlier report [5,29]. In our study area, footwear use is highly variable, particularly in rural communities. Although many individuals wear shoes, their use is often inconsistent, with footwear frequently removed during farming, household tasks, and outdoor activities. Children, in particular, commonly engage in outdoor play barefoot or with exposed legs, increasing direct contact with contaminated soil, the primary route of *strongyloidiasis* transmission. Moreover, based on our observations during data collection, most schoolchildren wore open slippers rather than closed shoes, which provide limited protection against exposure to contaminated soil.

The significant association between *S. stercoralis* infection and close contact with dogs was obtained in this study. This supports the growing evidence for a One Health perspective, where domestic animals may serve as reservoirs or mechanical carriers of infection. The present study report is supported by a previous study [24]. Although dog contact was associated with *S. stercoralis* infection in our study, the role of dogs as potential reservoir hosts cannot be confirmed because animal sampling and molecular characterization were not performed. The association may also reflect shared environmental exposures or behaviors.

The co-endemicity of *S. stercoralis* and hookworm (16.7%) further indicates shared transmission pathways and overlapping risk factors. However, their detection mainly by KK highlights differences in diagnostic suitability for different parasites. This report is consistent with a previous report that showed co-endemicity of *S. stercoralis* and hookworm infection [30]. Both parasites are transmitted through contact with contaminated soil, particularly in environments characterized by poor sanitation, open defecation, and inadequate use of protective footwear. These common ecological and behavioral factors facilitate their simultaneous circulation within endemic communities.

In the present study, hookworm was detected using both the KK technique and agar plate APC, whereas *S. stercoralis* was detected using the BCT and APC. This diagnostic combination is important for interpretation of co-endemicity. Kato-Katz is well suited for detecting hookworm eggs, while APC increases sensitivity by allowing larval development and recovery, thereby improving detection of hookworm infections. In contrast, *S. stercoralis* does not reliably produce eggs in stool, making KK unsuitable [31]; instead, BCT is specifically used to recover larvae from stool samples, and APC serves as a highly sensitive complementary method for both parasites. Thus, the use of different but complementary diagnostic tools ensures more accurate identification of each infection and reduces underestimation, particularly for *S. stercoralis* [7–9]. This finding is consistent with previous reports demonstrating the co-existence of *S. stercoralis* and hookworm infections in endemic areas [8,9,24,30], and it highlights the importance of using multiple diagnostic approaches to accurately assess true infection burden. It also supports integrated control strategies targeting STHs, given their shared transmission dynamics and overlapping epidemiological profiles.

Ethiopia has implemented several public health interventions to reduce the burden of soil-transmitted helminths, including school-based preventive chemotherapy, health education, and improvements in water, sanitation, and hygiene (WASH). These interventions have contributed to reductions in the prevalence and intensity of several soil-transmitted helminth infections in many parts of the country, including the Amhara Region [32]. However, *Strongyloides stercoralis* has received comparatively little attention because routine surveillance rarely includes sensitive diagnostic methods, and the commonly administered anthelmintic drugs, such as albendazole and mebendazole, have limited efficacy against this parasite. Consequently, *S. stercoralis* transmission may persist despite successful control of other soil-transmitted helminths [33]. The high burden of infection observed among household members in the present study highlights the need to incorporate *S. stercoralis* into national control strategies through improved diagnostics, targeted treatment with effective drugs such as ivermectin, strengthened WASH interventions, household-based surveillance, and One Health approaches that address potential zoonotic transmission.

This study has some limitations. Its cross-sectional design precludes establishing the temporal sequence or direction of *Strongyloides stercoralis* transmission among household members. Additionally, molecular characterization of parasite isolates was not performed, limiting assessment of transmission pathways. Absence of comparing households of *S. stercoralis*-infected schoolchildren with those of uninfected schoolchildren. Longitudinal studies incorporating molecular epidemiological approaches are needed to better understand transmission dynamics.

## Conclusion

The high prevalence of *Strongyloides stercoralis* among schoolchildren and among their household members, indicating familial clustering of infection. BCT and APC provide an improved sensitivity in *S. stercoralis* detection. Key risk factors, including living on mud flooring; close contact with dogs; a clay soil environment; irrigation activities; and inconsistent shoe wearing, aggravated the strongyloidiasis transmission in the community. Hence, these findings call for a comparison of integrated control strategies that combine improved diagnostics, targeted treatment of both schoolchildren and household members, health education, promotion of footwear use, and environmental sanitation. Future *One Health studies incorporating simultaneous sampling of humans, dogs, and environmental sources are recommended to clarify the role of dogs in the transmission dynamics of S. stercoralis*. In addition, future studies should include households of both infected and uninfected school-aged children to enable direct comparisons and provide stronger evidence on familial clustering and transmission dynamics.
