# Supplementary material for: Tracing Strongyloides stercoralis transmission and risk factors from schoolchildren to household in Northwest Ethiopia
Source: PLoS Negl Trop Dis. 2026 Nov 2;20(11):e0014857. doi: 10.1371/journal.pntd.0014857 (PMC13743027; doi:10.1371/journal.pntd.0014857)
Supplement: Supplementary file 1 [file pntd.0014857.s002.docx]

**Authors’ response to editor and reviewers’ comments**

**Authors’ response to editor’s and reviewers’ comments**

We sincerely thank the editor and the reviewers for their thoughtful, constructive, and insightful comments, which have significantly improved the quality and clarity of our manuscript. We have carefully considered every comment and revised the manuscript accordingly. A detailed point-by-point response has been provided for each comment, and all corresponding changes have been incorporated into the revised manuscript and highlighted in yellow for ease of reference. We hope that our revisions have adequately addressed all of the editor's and reviewers' concerns. If any further revisions is required, we would be grateful for the opportunity to provide them and further improve the manuscript.

**Editors comment**

If the reviewer comments include a recommendation to cite specific previously published works, please review and evaluate these publications to determine whether they are relevant and should be cited. There is no requirement to cite these works unless the editor has indicated otherwise.

**Authors’ response**: We thank the reviewer for the careful evaluation of our manuscript. Nevertheless, we carefully reviewed the relevant and recent literature during the revision process

1) We have amended your Competing Interest statement to comply with journal style. We kindly ask that you double check the statement and let us know if anything is incorrect.

**Authors’ response:** Thank you for amending the Competing Interests statement to comply with the journal style. We have carefully reviewed the revised statement and confirm that it is accurate. No further corrections are required.

**Reviewer's Responses to Questions**

**Key Review Criteria Required for Acceptance?**

**Methods**
-Are the objectives of the study clearly articulated with a clear testable hypothesis stated?

-Is the study design appropriate to address the stated objectives?

-Is the population clearly described and appropriate for the hypothesis being tested?

-Is the sample size sufficient to ensure adequate power to address the hypothesis being tested?

-Were correct statistical analysis used to support conclusions?

-Are there concerns about ethical or regulatory requirements being met?

**Reviewer #1: (No Response)**

**Reviewer #2:**

- To assess the prevalence of S. stercoralis in schoolchildren and identify infection pathways, stool samples were collected from 297 randomly selected schoolchildren from three primary schools in Bahir Dar City and the Bahir Dar Zuria district (Ethiopia). The stool was screened for the presence of S. stercoralis (and other helminths) using three diagnostic methods: the Baermann concentration technique (BCT), agar plate culture (APC), and the Kato-Katz technique. The latter was primarily used for the detection of helminths other than S. stercoralis.

**Authors’ response:** We thank the reviewer for providing this accurate summary of our study. We appreciate the reviewer's careful assessment of our work. No changes to the manuscript were required in response to this comment.

- Subsequently, stool samples were collected from household members of schoolchildren who tested positive for S. stercoralis infection. Household members of students who did not test positive for S. stercoralis were not screened. Schoolchildren and family members who were taking anthelmintic drugs were excluded from the study.
- **Authors’ response:** We thank the reviewer for accurately summarizing our study design. As noted, only household members of schoolchildren who tested positive for Strongyloides stercoralis were included to investigate household transmission and potential infection pathways from index cases. Schoolchildren and household members who had received anthelmintic treatment during the study period were excluded to avoid the influence of recent treatment on parasite detection.
- Socio-demographic characteristics and risk factors were collected from study participants. The questionnaire is not provided with the manuscript, however the criteria are listed in tables and text. While one criteria is mentioned as “contact with domestic animals” in the methods section and in the discussion, in the results it is specified as “having close contact to dogs”. Stool samples of the dogs, which are reservoir hosts for S. stercoralis, have not been included in this study.
  **Authors’ response:** We sincerely thank the reviewer for this important comment. We agree that our study did not include stool sampling from dogs or molecular characterization of Strongyloides stercoralis isolates. This was not part of the study objectives, which focused on determining the prevalence of S. stercoralis among school-aged children and their household members and identifying associated risk factors. Accordingly, we have moderated our discussion regarding the potential role of dogs in transmission. We now clarify that the observed association between dog contact and human infection should be interpreted with caution, as our findings cannot determine whether dogs serve as reservoir hosts or whether the association reflects shared environmental exposures. We have addressed this point in the Recommendations section of the revised manuscript by stating (line 394-94).
- The dataset is thus highly skewed towards S. stercoralis positive participants and numbers do not reflect the “total prevalence” or “true prevalence” correctly. Only examining family members of S. stercoralis positive children and calculating prevalence by using the combined numbers of both schoolchildren and household member samples is a grave statistical, methodical, and logical error. When wanting to assess risk factors for infection and the impact of infected family members on the infection status of the child, investigating the families of S. stercoralis negative children would have been equally informative.

**Authors’ response:** We sincerely thank the reviewer for this important methodological comment. We agree that household members were not randomly selected from the general population but were recruited exclusively from households of *Strongyloides stercoralis*-positive schoolchildren to investigate household clustering and potential transmission. Therefore, revision has been done (line 202-209).
It is not stated in the manuscript if all stool samples were screened with all three techniques. As stated by the authors, each method provided additional positive cases. The amount of S. stercoralis positive samples detected varied significantly between methods, the detection success rates between the techniques also varied greatly between sample sets.

Differences in detection success between diagnostic methods APC and BCT are common, however in the case of the presented results, these differences are rather striking: for a total of 116 positive samples, 70 (~60% of 116) were positive by BTC and 78 (~67% of 116) by APC. If my calculations are correct, this means that only 32 out of 116 infections (~27.6) were confirmed by both methods. Compared to similar studies, this is unusual and the discrepancy and low detection success (especially by the otherwise rather reliable APC) requires further explanation/ discussion.

**Authors’ response:** We sincerely thank the reviewer for this insightful comment. We have revised the Methods section to explicitly state that all stool samples collected from both schoolchildren and household members were examined using all three diagnostic techniques. Revision has been done as per recommendation in the method (line 123-125) and result (208-211) sections.

- Unfortunately the study does not read methodically sound at this stage. Specific information about the study setup (choice and definition of criteria, choice of participants, all stool samples screened with all techniques or varied) is lacking. Statistical analysis and interpretation needs to be reassessed.

**Authors’ response:** We sincerely thank the reviewer for the thorough and constructive evaluation of our manuscript. We appreciate the concerns raised regarding the methodological description, study design, and statistical interpretation. In response, we have carefully revised the manuscript to improve its clarity, transparency, and methodological rigor. Specifically, we have clarified that the three primary schools were selected using a random sampling procedure. Within each selected school, students were randomly selected, and the sample size was proportionally allocated according to the student population of each school. These methodological details have been clearly described in the revised Methods section (line 106-116).

**Reviewer #3: Yes**

**Authors’ response:** Thank you for your positive assessment.

**Results**
-Does the analysis presented match the analysis plan?

-Are the results clearly and completely presented?

-Are the figures (Tables, Images) of sufficient quality for clarity?

**Reviewer #1: (No Response)**

**Authors’ response:** We thank for evaluating our manuscript. As no specific comments or suggestions were provided. Revisions of the result section has been done based on other reviews.

**Reviewer #2:** The Results chapter is written in a rather confusing manner, it contains inconsistencies and many repetitions.

It also contains various semantic and numerical errors. Some examples only for the section before Table 1 are:
Line 192: 123 male participants are listed in the text, but there are 134 elsewhere, which seems to be the correct number given the total participant number.

Line 193: “The proportion of female participants was in 1:1 ratio with that of females.” – this is nonsensical. If it is supposed to mean “a 1:1 ratio of female to male participants”, this is also not true.

Line 195: There is a typo, the age bracket is 9-11, not 19-11.

Line 198: The text states that females accounted for 58.8% of family members, in table 1 this is the percentage of males.

Other than, the issues regarding prevalence calculations and statistics (as already mentioned regarding the methods chapter), the results require streamlining to reduce repetition, as well as error correction.

**Authors’ response:** We sincerely thank the reviewer for the careful evaluation of the results section and for identifying these important inconsistencies. We have thoroughly revised the results to improve clarity, accuracy, and readability by eliminating unnecessary repetition, correcting typographical and numerical errors, and ensuring consistency between the text and tables (line 195-203).
**Reviewer #3: Yes**

**Authors’ response:** We sincerely thank for the positive evaluation of our manuscript. We appreciate the reviewer's assessment that the analyses were consistent with the study objectives, that the results were clearly and comprehensively presented, and that the tables and figures were of sufficient quality and clarity. We are grateful for the reviewer's time and valuable evaluation.

**Conclusions**
-Are the conclusions supported by the data presented?

-Are the limitations of analysis clearly described?

-Do the authors discuss how these data can be helpful to advance our understanding of the topic under study?
-Is public health relevance addressed?

**Reviewer #1: (No Response)**

**Authors’ response:** We sincerely thank Reviewer #1 for the careful evaluation of our manuscript. We appreciate the reviewer's assessment and the time devoted to reviewing our work.

**Reviewer #2:** The conclusions drawn in the discussion are very general in terms of content and there are many repetitions throughout the chapter, sometimes even in consecutive sentences (l. 293-296).
The discussion would profit from a more in depth analysis of the data collected in this study, to highlight its relevance. While some comparisons with results of previous studies have been made, the differences in study setup and study groups are not discussed in detail to provide an accurate context.
Thus, the conclusions drawn in the discussion are mostly reinforced by the previous studies cited; the data collected in the present study does not add a lot of novel information, mainly because of the flawed and incompletely presented methodology. Reassessing how the data is evaluated could help substantiate its worth, especially in the context of similar studies conducted in Ethiopia throughout the years.
**Authors’ response:** We sincerely thank the reviewer for this thoughtful and constructive comment. In response, we have substantially revised the conclusion to improve its scientific depth, clarity, and coherence (line 401-412).

**Reviewer #3: Yes**

**Authors’ response:** We sincerely thank the reviewer for the positive assessment of the Conclusions section.

**Editorial and Data Presentation Modifications?**

Use this section for editorial suggestions as well as relatively minor modifications of existing data that would enhance clarity. If the only modifications needed are minor and/or editorial, you may wish to recommend “Minor Revision” or “Accept”.

**Reviewer #1: (No Response)**

**Authors’ response:** We thank the reviewer for the careful evaluation of our manuscript. No specific editorial or data presentation comments were provided in this section. However, we have thoroughly reviewed the manuscript and made necessary minor editorial corrections, improved clarity of text, and refined the presentation of data where appropriate to enhance the overall quality and readability of the manuscript.

**Reviewer #2: (No Response)**

**Authors’ response:** We thank the reviewer for the careful evaluation of our manuscript. No specific editorial or data presentation comments were provided in this section. However, we have thoroughly reviewed the manuscript and made necessary minor editorial corrections, improved clarity of text, and refined the presentation of data where appropriate to enhance the overall quality and readability of the manuscript.

**Reviewer #3: (No Response)**

**Authors’ response:** We thank the reviewer for the careful evaluation of our manuscript. No specific editorial or data presentation comments were provided in this section. However, we have thoroughly reviewed the manuscript and made necessary minor editorial corrections, improved clarity of text, and refined the presentation of data where appropriate to enhance the overall quality and readability of the manuscript.

**Summary and General Comments**

Use this section to provide overall comments, discuss strengths/weaknesses of the study, novelty, significance, general execution and scholarship. You may also include additional comments for the author, including concerns about dual publication, research ethics, or publication ethics. If requesting major revision, please articulate the new experiments that are needed.

**Reviewer #1:** In this paper, the authors conducted a screening and questionnaire survey on Strongyloides stercoralis infection among school-aged children and their families in three schools in northwestern Ethiopia between 2024 and 2025. The results showed significant clustering of infection within affected families. This topic is important because it highlights the urgency of developing integrated prevention and control strategies, including family-level screening and treatment, in northwestern Ethiopia, particularly in areas where awareness of S. stercoralis remains insufficient. However, the manuscript is not yet ready for publication, and some issues require revision to better reflect the limitations of the study design. These issues are discussed below.

**Major Comments**

1. This study is a cross-sectional study, assessing infection status only at a single time point. Although a high rate of Strongyloides stercoralis infection was observed among family members of infected school-aged children, the data did not determine the direction or timing of transmission. While existing evidence suggests familial clustering of S. stercoralis infection in the region, the results support familial clustering but not direct familial transmission. The authors' current study design cannot directly prove familial transmission, and the conclusion that transmission occurs from school-aged children to family members should be interpreted with caution and greater care.

**Authors’ response:** We sincerely thank the reviewer for this important and constructive comment. We agree that, due to the cross-sectional design of our study, the temporal sequence and directionality of S. stercoralis transmission cannot be established. Specifically, we have replaced statements suggesting that school-aged children were the source of transmission with more cautious wording indicating that the observed pattern supports **familial clustering of *S. stercoralis* infection** and suggests potential shared environmental, behavioral, or household-related risk factors. We have also expanded the limitations section to explicitly acknowledge that longitudinal studies with molecular epidemiological approaches are required to determine transmission pathways and temporal relationships among household members (line 395-399).
2. Close contact with dogs is considered an important risk factor. However, this study did not perform animal sampling or molecular typing or genotyping. Therefore, this study cannot determine whether dogs serve as reservoir hosts or simply represent markers of shared environmental exposure. The evidence supporting zoonotic transmission is limited, and this limitation should be clearly stated in the discussion section.

**Authors’ response:** We sincerely thank the reviewer for highlighting this important point. We agree that, although close contact with dogs has been reported as a potential risk factor for S stercoralis infection. However, the primary objective of our study was to trace S. stercoralis infection among household members of infected school-aged children and to identify potential household- and behavior-related risk factors associated with transmission. We did not include animal sampling, molecular characterization, or genotyping of parasite isolates for this study due to budget constrained. In any case, we have revised the discussion part as a limitation (line 195-199).

3. The association between wearing shoes and increased infection risk appears inconsistent with known S. stercoralis transmission biology. Providing more information on how to measure and classify shoe-wearing behavior will help readers understand this finding. Furthermore, potential residual confounding risk factors should be considered and further explanation is needed.

**Authors’ response:** We sincerely thank the reviewer for this insightful comment. We agree that the observed association between shoe-wearing behavior and *S. stercoralis* infection requires careful interpretation. In response, we have revised the discussion section to provide a clearer description of how shoe-wearing behavior was assessed and classified based on participants’ self-reported habitual footwear use during the questionnaire survey. We have also expanded the discussion to provide context for this finding. In our study area, particularly in rural communities, footwear use is highly variable (line 345-352)

4. Family members only come from households with infected school-aged children. Including households with uninfected school-aged children would provide a more appropriate control group and better explain family clustering.

**Authors’ response:** We sincerely thank the reviewer for this valuable comment. We agree that including households of uninfected school-aged children would have provided an appropriate comparison group and strengthened the assessment of familial clustering. However, the primary objective of this study was to trace S. stercoralis infection among household members of infected school-aged children and to identify potential household- and behavior-related risk factors associated with infection. Accordingly, only households of infected index children were included in the study design. We revised and indicated in the recommendation (410-412)

5. The data from authors showed the prevalence among household members is remarkably high; however, only 23 of 62 index-positive children's households participated in household screening. They should discuss the potential impact of participation bias and whether participating households differed from those that did not participate. Additionally, the authors should explicitly define how infection status was determined when results from BCT and APC were discordant.

**Authors’ response: Authors’ response:** We sincerely thank the reviewer for this valuable comment. All households of *S. stercoralis*-infected school-aged children were approached using the same recruitment procedures and were provided with identical information regarding the purpose and procedures of the study. Therefore, we did not anticipate systematic differences in recruitment or participation arising from the information provided. Nevertheless, because only 23 of the 62 eligible households participated, we acknowledge that some degree of participation bias cannot be entirely excluded. We have also clarified the definition of infection status in the Methods section (line 123-125).

**Reviewer #2: (No Response)**

**Authors’ response:** We sincerely thank the reviewer for the careful evaluation of our manuscript. No additional comments were provided in this section.

**Reviewer #3**: Reviewers’ comments

This is an important paper because strongyloidiasis remains a public health concern in Africa, and identifying its risk factors can inform disease control programs and guide public health interventions.
**Comments**
Line 117: To estimate your sample size, you used a design effect of 1.5. Could you provide any reference for that?

**Authors’ response:** We sincerely thank the reviewer for the careful evaluation of our manuscript. Revision has been done (line 119)

Line 118 – 121: You only considered 3 schools without clearly mentioning whether the number of children per school, was it the same or proportional to the size of the sampling unit?

**Authors’ response:** We sincerely thank the reviewer for the careful evaluation. Proportional allocation was done and indicated in the revised manuscript (109-111).

Line 173: you mentioned that you used appropriate software, please can you be more specific by providing the exact software name and its version. For instance, R version 4.5 or Stata version 15
**Authors’ response**: Thank you for your comments. Revision has been done (line 178-79)
Line 174: You mentioned you summarized the prevalence of S. stercoralis. I think you should mention clearly that on top of that you described the socio-demographic parameters as we can see in your Table 1. You should describe how you categorised some of the qualitative variable such as age group etc.
**Authors’ response:** Thank you for your comments. Revision has been done in the method section (line 128-131)
Line 235: I propose you replace address by residence

**Authors’ response:** Thank you for your comments. Revision has been done as per the recommendation in the tables.

Line 270: Univariate and multivariate analysis of factors associated with S. stercoralis infection among family members across their demographic factors in Northwest Ethiopia. When looking at the number of observations used for this model, you mentioned 273, meaning that it is for school-age children and not among family members. In addition, do you have similar table for family members (80 in total)?.
**Authors’ response:** We sincerely thank the reviewer for carefully identifying this inconsistency. The reviewer is correct that the analyses presented in Table 4 were based on 273 school-aged children and not on household members. The phrase "among family members" in the table title was therefore incorrect and has been revised to "among school-aged children" in the updated manuscript. Regarding household members, the sample size (n = 80) was relatively small, limiting the statistical power for a reliable regression analysis. Therefore, we did not perform a separate multivariable analysis for household members. We have clarified this in the revised manuscript to avoid any misunderstanding.

Overall comment: you mentioned in your manuscript title: Tracing S. stercoralis and risk factors. I think you mainly focus on identifying the risk factors. If you think the fact of going to children family members is tracing the transmission, you make this explicitly clear in the introduction, and discussion.

**Authors’ response:** We sincerely thank the reviewer for this insightful comment. We agree that the concept of "tracing" should be more clearly defined in the manuscript. Our intention was not to imply direct tracing of transmission pathways, but rather to trace *Strongyloides stercoralis* infection by screening household members of infected school-aged children and to identify potential household- and behavior-related risk factors associated with infection. In response, we have revised the introduction to explicitly state this objective and have strengthened the discussion to clarify that our findings provide evidence of familial clustering rather than direct transmission.
