# Supplementary material for: Tracing Strongyloides stercoralis transmission and risk factors from schoolchildren to household in Northwest Ethiopia
Source: PLoS Negl Trop Dis. 2026 Nov 2;20(11):e0014857. doi: 10.1371/journal.pntd.0014857 (PMC13743027; doi:10.1371/journal.pntd.0014857)
Supplement: Supplementary file 2 [file pntd.0014857.s003.docx]

**Response to Reviewers**

**Authors’ response to editor’s and reviewers’ comments**

We sincerely thank the editor and the reviewers for their thoughtful, constructive, and insightful comments, which have significantly improved the quality and clarity of our manuscript. We have carefully considered every comment and revised the manuscript accordingly. A detailed point-by-point response has been provided for each comment, and all corresponding changes have been incorporated into the revised manuscript and highlighted in yellow for ease of reference. We hope that our revisions have adequately addressed all of the editors’ and reviewers' concerns. If any further revisions is required, we would be very happy to review again to improve the manuscript.

**Reviewers' comments:**

 Reviewer's Responses to Questions

**Key Review Criteria Required for Acceptance?**

As you describe the new analyses required for acceptance, please consider the following:
**Methods**

- Are the objectives of the study clearly articulated with a clear testable hypothesis stated?
- -Is the study design appropriate to address the stated objectives?
- -Is the population clearly described and appropriate for the hypothesis being tested?
- -Is the sample size sufficient to ensure adequate power to address the hypothesis being tested?
- -Were correct statistical analysis used to support conclusions?
- -Are there concerns about ethical or regulatory requirements being met?

 Reviewer #1: (No Response)

Reviewer #3: Yes

**Authors’ response**: We sincerely thank reviewer #3 for the positive assessment of our manuscript and for confirming that the study meets the key review criteria required for acceptance. We appreciate the reviewer’s evaluation of the clarity of the study objectives and hypothesis, appropriateness of the study design and population, adequacy of the sample size, validity of the statistical analyses, and compliance with ethical and regulatory requirements. We have carefully reviewed the manuscript to ensure that these aspects are clearly presented and adequately addressed. We believe that the manuscript provides a sound methodological basis for the study findings and conclusions.

**Results**

- Does the analysis presented match the analysis plan?
- Are the results clearly and completely presented?
- Are the figures (Tables, Images) of sufficient quality for clarity?
- Reviewer #1: (No Response)
- Reviewer #3: Yes

**Authors’ response:** We sincerely thank Reviewer #3 for the positive assessment of the Results section. We appreciate the reviewer’s confirmation that the analyses presented are consistent with the analysis plan, the results are clearly and completely presented, and the tables and figures are of sufficient quality to ensure clarity. We have reviewed the Results section to maintain consistency between the planned analyses, reported findings, and study objectives. We believe that the presentation of the results adequately supports the study conclusions.

**Conclusions**

- Are the conclusions supported by the data presented?
- Are the limitations of analysis clearly described?
- Do the authors discuss how these data can be helpful to advance our understanding of the topic under study?
- Is public health relevance addressed?

Reviewer #1: The authors have made substantial revisions to the manuscript and have addressed most of the concerns raised during the previous review. However, a few issues still require clarification or correction before the manuscript can be considered for acceptance.
1. The major concern regarding the interpretation of household transmission has been partially addressed. The authors now appropriately acknowledge that longitudinal and molecular epidemiological studies are required to determine transmission pathways. However, statements such as “ongoing household transmission” and “sustained intra-household transmission” remain stronger than supported by the cross-sectional study design. The current data support household or familial clustering of infection but cannot establish direct transmission or its directionality. Please ensure that this distinction is consistently reflected throughout the Abstract, Authors’ Summary, and Conclusion.

**Authors’ response:** We sincerely thank the reviewer #1 for this valuable comment. We agree that the cross-sectional design of our study does not allow us to establish direct household transmission, determine the directionality of transmission, or confirm ongoing or sustained intra-household transmission. Our findings demonstrate household or familial clustering of infection rather than direct evidence of transmission between household members. Revision has been done throughout the document. (line 49, & 403)

2. The authors have appropriately acknowledged the possibility of participation bias because only 23 of the 62 eligible households participated in household screening. However, it remains unclear whether the characteristics of participating and non-participating households could be compared. If such data are unavailable, this should be explicitly stated, and the potential impact of participation bias on the estimated household prevalence and its generalizability should be clearly acknowledged.

**Authors’ response:** We sincerely thank Reviewer #1 for highlighting this important methodological limitation. The primary objective of household screening was to assess Strongyloides stercoralis infection among family members of infected schoolchildren, rather than to compare households of infected and uninfected schoolchildren. Of the 62 eligible households, only 23 participated in household screening. We acknowledge that information on participating and non-participating households was unavailable for comparison; therefore, we could not assess potential differences between these groups. This may have introduced participation bias, potentially resulting in overestimation or underestimation of household infection prevalence and limiting the generalizability of the findings. We have explicitly acknowledged this limitation in the revised manuscript (line 398-99 & 411-413).

Reviewer #3: Yes

**Authors’ response:** We sincerely thank Reviewer #3 for the positive assessment of the Conclusions section. We appreciate the reviewer’s confirmation that the conclusions are supported by the data, the limitations are adequately described, the contribution of the findings to advancing understanding of the study topic is addressed, and the public health relevance is clearly presented. We have also carefully considered the additional clarifications requested by Reviewer #1 and refined the relevant statements to ensure that the conclusions remain consistent with the cross-sectional study design and acknowledge the potential impact of participation bias.

**Editorial and Data Presentation Modifications?**

Use this section for editorial suggestions as well as relatively minor modifications of existing data that would enhance clarity. If the only modifications needed are minor and/or editorial, you may wish to recommend “Minor Revision” or “Accept”.

Reviewer #1: (No Response)

Reviewer #3: (No Response)

**Authors’ response:** We thank the reviewers for their careful evaluation of our manuscript. No specific editorial or data presentation modifications were raised under this section. Nevertheless, we have carefully reviewed the manuscript to improve clarity, consistency, and presentation, including ensuring that statements regarding household infection clustering and the potential for participation bias accurately reflect the study design and available data.

**Summary and General Comments**

Use this section to provide overall comments, discuss strengths/weaknesses of the study, novelty, significance, general execution and scholarship. You may also include additional comments for the author, including concerns about dual publication, research ethics, or publication ethics. If requesting major revision, please articulate the new experiments that are needed.

Reviewer #1: The authors have made substantial revisions to the manuscript and have addressed most of the concerns raised during the previous review. However, a few issues still require clarification or correction before the manuscript can be considered for acceptance.
1. The major concern regarding the interpretation of household transmission has been partially addressed. The authors now appropriately acknowledge that longitudinal and molecular epidemiological studies are required to determine transmission pathways. However, statements such as “ongoing household transmission” and “sustained intra-household transmission” remain stronger than supported by the cross-sectional study design. The current data support household or familial clustering of infection but cannot establish direct transmission or its directionality. Please ensure that this distinction is consistently reflected throughout the Abstract, Authors’ Summary, and Conclusion.

**Authors’ response:** We sincerely thank Reviewer #1 for recognizing the substantial revisions made to our manuscript and acknowledging that most of the concerns raised during the previous review have been addressed. Revision has been done as per your recommendation above (line 49 & 403)

2. The authors have appropriately acknowledged the possibility of participation bias because only 23 of the 62 eligible households participated in household screening. However, it remains unclear whether the characteristics of participating and non-participating households could be compared. If such data are unavailable, this should be explicitly stated, and the potential impact of participation bias on the estimated household prevalence and its generalizability should be clearly acknowledged.

**Authors’ response:** We sincerely thank the reviewer for highlighting the potential impact of participation bias on our household-level findings. Revision has been done as per your comments above (line 398-99 & 411-413)

Reviewer #3: The authors have addressed my all previous comments.

**Authors response:** We sincerely thank Reviewer #3 for carefully reviewing our revised manuscript and for confirming that all previous comments have been addressed. We greatly appreciate the reviewer’s constructive feedback and valuable suggestions throughout the review process, which have helped us improve the clarity, scientific rigor, and overall quality of our manuscript.

**Figure resubmission:**

While revising your submission, we strongly recommend that you use PLOS’s NAAS tool (<https://ngplosjournals.pagemajik.ai/artanalysis>) to test your figure files. NAAS can convert your figure files to the TIFF file type and meet basic requirements (such as print size, resolution), or provide you with a report on issues that do not meet our requirements and that NAAS cannot fix.

After uploading your figures to PLOS’s NAAS tool  [https://ngplosjournals.pagemajik.ai/artanalysis](https://ngplosjournals.pagemajik.ai/artanalysis), NAAS will process the files provided and display the results in the "Uploaded Files" section of the page as the processing is complete. If the uploaded figures meet our requirements (or NAAS is able to fix the files to meet our requirements), the figure will be marked as "fixed" above. If NAAS is unable to fix the files, a red "failed" label will appear above. When NAAS has confirmed that the figure files meet our requirements, please download the file via the download option, and include these NAAS processed figure files when submitting your revised manuscript.

**Authors’ response:** Thank you for the instructions regarding figure resubmission. Our manuscript does not contain any figures; therefore, figure processing using the PLOS NAAS tool is not applicable to our submission.
