# Supplementary material for: Tracing Strongyloides stercoralis transmission and risk factors from schoolchildren to household in Northwest Ethiopia
Source: PLoS Negl Trop Dis. 2026 Nov 2;20(11):e0014857. doi: 10.1371/journal.pntd.0014857 (PMC13743027; doi:10.1371/journal.pntd.0014857)
Supplement: Peer review history

# Peer review: Decision Letter 0

**DOI:** 10.1371/journal.pntd.0014857.r001

**Authors:** Peter Fischer, Hugues Nana Djeunga

14 Jul 2026

PNTD-D-26-00780

Tracing Strongyloides stercoralis Transmission and Risk Factors from Schoolchildren to Household in Northwest Ethiopia

PLOS Neglected Tropical Diseases

Dear Dr. Hailu,

Thank you for submitting your manuscript to PLOS Neglected Tropical Diseases. After careful consideration, we feel that it has merit but does not fully meet PLOS Neglected Tropical Diseases's publication criteria as it currently stands. Therefore, we invite you to submit a revised version of the manuscript that addresses the points raised during the review process.

Please submit your revised manuscript within by Sep 12 2026 11:59PM. If you will need more time than this to complete your revisions, please reply to this message or contact the journal office at plosntds@plos.org. When you're ready to submit your revision, log on to https://www.editorialmanager.com/pntd/ and select the 'Submissions Needing Revision' folder to locate your manuscript file.

Please include the following items when submitting your revised manuscript:

* A letter that responds to each point raised by the editor and reviewer(s). You should upload this letter as a separate file labeled 'Response to Reviewers'. This file does not need to include responses to any formatting updates and technical items listed in the 'Journal Requirements' section below.

* A marked-up copy of your manuscript that highlights changes made to the original version. You should upload this as a separate file labeled 'Revised Manuscript with Track Changes'.

* An unmarked version of your revised paper without tracked changes. You should upload this as a separate file labeled 'Manuscript'.

If you would like to make changes to your financial disclosure, competing interests statement, or data availability statement, please make these updates within the submission form at the time of resubmission. Guidelines for resubmitting your figure files are available below the reviewer comments at the end of this letter.

As the corresponding author, your ORCID iD is verified in the submission system and will appear in the published article. PLOS supports the use of ORCID, and we encourage all coauthors to register for an ORCID iD and use it as well. Please encourage your coauthors to verify their ORCID iD within the submission system before final acceptance, as unverified ORCID iDs will not appear in the published article. Only the individual author can complete the verification step; PLOS staff cannot verify ORCID iDs on behalf of authors.

We look forward to receiving your revised manuscript.

Kind regards,

Hugues C. Nana Djeunga, PhD

Academic Editor

PLOS Neglected Tropical Diseases

Peter Fischer

Section Editor

PLOS Neglected Tropical Diseases

Shaden Kamhawi

co-Editor-in-Chief

PLOS Neglected Tropical Diseases

orcid.org/0000-0003-4304-636XX

Paul Brindley

co-Editor-in-Chief

PLOS Neglected Tropical Diseases

orcid.org/0000-0003-1765-0002

**Additional Editor Comments (if provided):**

**Journal Requirements:**

If the reviewer comments include a recommendation to cite specific previously published works, please review and evaluate these publications to determine whether they are relevant and should be cited. There is no requirement to cite these works unless the editor has indicated otherwise.

1) We have amended your Competing Interest statement to comply with journal style. We kindly ask that you double check the statement and let us know if anything is incorrect.

**Reviewers' Comments:**

Reviewer's Responses to Questions

**Key Review Criteria Required for Acceptance?**

As you describe the new analyses required for acceptance, please consider the following:

**Methods**

-Are the objectives of the study clearly articulated with a clear testable hypothesis stated?

-Is the study design appropriate to address the stated objectives?

-Is the population clearly described and appropriate for the hypothesis being tested?

-Is the sample size sufficient to ensure adequate power to address the hypothesis being tested?

-Were correct statistical analysis used to support conclusions?

-Are there concerns about ethical or regulatory requirements being met?

Reviewer #1: (No Response)

Reviewer #2: To assess the prevalence of S. stercoralis in schoolchildren and identify infection pathways, stool samples were collected from 297 randomly selected schoolchildren from three primary schools in Bahir Dar City and the Bahir Dar Zuria district (Ethiopia). The stool was screened for the presence of S. stercoralis (and other helminths) using three diagnostic methods: the Baermann concentration technique (BCT), agar plate culture (APC), and the Kato-Katz technique. The latter was primarily used for the detection of helminths other than S. stercoralis.

Subsequently, stool samples were collected from household members of schoolchildren who tested positive for S. stercoralis infection. Household members of students who did not test positive for S. stercoralis were not screened. Schoolchildren and family members who were taking anthelmintic drugs were excluded from the study.

Socio-demographic characteristics and risk factors were collected from study participants. The questionnaire is not provided with the manuscript, however the criteria are listed in tables and text. While one criteria is mentioned as “contact with domestic animals” in the methods section and in the discussion, in the results it is specified as “having close contact to dogs”. Stool samples of the dogs, which are reservoir hosts for S. stercoralis, have not been included in this study.

The dataset is thus highly skewed towards S. stercoralis positive participants and numbers do not reflect the “total prevalence” or “true prevalence” correctly. Only examining family members of S. stercoralis positive children and calculating prevalence by using the combined numbers of both schoolchildren and household member samples is a grave statistical, methodical, and logical error. When wanting to assess risk factors for infection and the impact of infected family members on the infection status of the child, investigating the families of S. stercoralis negative children would have been equally informative.

It is not stated in the manuscript if all stool samples were screened with all three techniques. As stated by the authors, each method provided additional positive cases. The amount of S. stercoralis positive samples detected varied significantly between methods, the detection success rates between the techniques also varied greatly between sample sets.

Differences in detection success between diagnostic methods APC and BCT are common, however in the case of the presented results, these differences are rather striking: for a total of 116 positive samples, 70 (~60% of 116) were positive by BTC and 78 (~67% of 116) by APC. If my calculations are correct, this means that only 32 out of 116 infections (~27.6) were confirmed by both methods. Compared to similar studies, this is unusual and the discrepancy and low detection success (especially by the otherwise rather reliable APC) requires further explanation/ discussion.

Unfortunately the study does not read methodically sound at this stage. Specific information about the study setup (choice and definition of criteria, choice of participants, all stool samples screened with all techniques or varied,…) is lacking. Statistical analysis and interpretation needs to be reassessed.

Reviewer #3: Yes

**********

**Results**

-Does the analysis presented match the analysis plan?

-Are the results clearly and completely presented?

-Are the figures (Tables, Images) of sufficient quality for clarity?

Reviewer #1: (No Response)

Reviewer #2: The Results chapter is written in a rather confusing manner, it contains inconsistencies and many repetitions.

It also contains various semantic and numerical errors. Some examples only for the section before Table 1 are:

Line 192: 123 male participants are listed in the text, but there are 134 elsewhere, which seems to be the correct number given the total participant number.

Line 193: “The proportion of female participants was in 1:1 ratio with that of females.” – this is nonsensical. If it is supposed to mean “a 1:1 ratio of female to male participants”, this is also not true.

Line 195: There is a typo, the age bracket is 9-11, not 19-11.

Line 198: The text states that females accounted for 58.8% of family members, in table 1 this is the percentage of males.

Other than the issues regarding prevalence calculations and statistics (as already mentioned regarding the methods chapter), the results require streamlining to reduce repetition, as well as error correction.

Reviewer #3: Yes

**********

**Conclusions**

-Are the conclusions supported by the data presented?

-Are the limitations of analysis clearly described?

-Do the authors discuss how these data can be helpful to advance our understanding of the topic under study?

-Is public health relevance addressed?

Reviewer #1: (No Response)

Reviewer #2: The conclusions drawn in the discussion are very general in terms of content and there are many repetitions throughout the chapter, sometimes even in consecutive sentences (l. 293-296).

The discussion would profit from a more in depth analysis of the data collected in this study, to highlight its relevance. While some comparisons with results of previous studies have been made, the differences in study setup and study groups are not discussed in detail to provide an accurate context.

Thus, the conclusions drawn in the discussion are mostly reinforced by the previous studies cited; the data collected in the present study does not add a lot of novel information, mainly because of the flawed and incompletely presented methodology. Reassessing how the data is evaluated could help substantiate its worth, especially in the context of similar studies conducted in Ethiopia throughout the years.

The impact of public health meaures, especially in the context of regional differences and changes in helminth prevalence over the years, is not adequately considered and discussed. A well researched section about public health measures in the Bahir Dar region could raise the quality of this manuscript significantly.

Reviewer #3: Yes

**********

**Editorial and Data Presentation Modifications?**

Use this section for editorial suggestions as well as relatively minor modifications of existing data that would enhance clarity. If the only modifications needed are minor and/or editorial, you may wish to recommend “Minor Revision” or “Accept”.

Reviewer #1: (No Response)

Reviewer #2: (No Response)

Reviewer #3: (No Response)

**********

**Summary and General Comments**

Use this section to provide overall comments, discuss strengths/weaknesses of the study, novelty, significance, general execution and scholarship. You may also include additional comments for the author, including concerns about dual publication, research ethics, or publication ethics. If requesting major revision, please articulate the new experiments that are needed.

Reviewer #1: In this paper, the authors conducted a screening and questionnaire survey on Strongyloides stercoralis infection among school-aged children and their families in three schools in northwestern Ethiopia between 2024 and 2025. The results showed significant clustering of infection within affected families. This topic is important because it highlights the urgency of developing integrated prevention and control strategies, including family-level screening and treatment, in northwestern Ethiopia, particularly in areas where awareness of S. stercoralis remains insufficient. However, the manuscript is not yet ready for publication, and some issues require revision to better reflect the limitations of the study design. These issues are discussed below.

Major Comments

1. This study is a cross-sectional study, assessing infection status only at a single time point. Although a high rate of Strongyloides stercoralis infection was observed among family members of infected school-aged children, the data did not determine the direction or timing of transmission. While existing evidence suggests familial clustering of S. stercoralis infection in the region, the results support familial clustering but not direct familial transmission. The authors' current study design cannot directly prove familial transmission, and the conclusion that transmission occurs from school-aged children to family members should be interpreted with caution and greater care.

2. Close contact with dogs is considered an important risk factor. However, this study did not perform animal sampling or molecular typing or genotyping. Therefore, this study cannot determine whether dogs serve as reservoir hosts or simply represent markers of shared environmental exposure. The evidence supporting zoonotic transmission is limited, and this limitation should be clearly stated in the discussion section.

3. The association between wearing shoes and increased infection risk appears inconsistent with known S. stercoralis transmission biology. Providing more information on how to measure and classify shoe-wearing behavior will help readers understand this finding. Furthermore, potential residual confounding risk factors should be considered and further explanation is needed.

4. Family members only come from households with infected school-aged children. Including households with uninfected school-aged children would provide a more appropriate control group and better explain family clustering.

5. The data from authors showed the prevalence among household members is remarkably high; however, only 23 of 62 index-positive children's households participated in household screening. They should discuss the potential impact of participation bias and whether participating households differed from those that did not participate. Additionally, the authors should explicitly define how infection status was determined when results from BCT and APC were discordant.

Reviewer #2: (No Response)

Reviewer #3: Reviewers’ comments

This is an important paper because strongyloidiasis remains a public health concern in Africa, and identifying its risk factors can inform disease control programs and guide public health interventions.

Comments

Line 117: To estimate your sample size, you used a design effect of 1.5. could you provide any reference for that?

Line 118 – 121: You only considered 3 schools without clearly mentioning whether the number of children per school, was it the same or proportional to the size of the sampling unit?

Line 173: you mentioned that you used appropriate software, please can you be more specific by providing the exact software name and its version. For instance, R version 4.5 or Stata version 15

Line 174: You mentioned you summarized the prevalence of S. stercoralis. I think you should mention clearly that on top of that you described the socio-demographic parameters as we can see in your Table 1. You should describe how you categorised some of the qualitative variable such as age group etc.

Line 235: I propose you replace address by residence

Line 270: Univariate and multivariate analysis of factors associated with S. stercoralis infection among family members across their demographic factors in Northwest Ethiopia. When looking at the number of observations used for this model, you mentioned 273, meaning that it is for school-age children and not among family members. In addition, do you have similar table for family members (80 in total)?.

Overall comment: you mentioned in your manuscript title: Tracing strongyloides stercoralis and risk factors. I think you mainly focus on identifying the risk factors. If you think the fact of going to children family members is tracing the transmission, you make this explicitly clear in the introduction, and discussion.

**********

PLOS authors have the option to publish the peer review history of their article (what does this mean?). If published, this will include your full peer review and any attached files.

If you choose “no”, your identity will remain anonymous but your review may still be made public.

**Do you want your identity to be public for this peer review?**  For information about this choice, including consent withdrawal, please see our Privacy Policy.

Reviewer #1: No

Reviewer #2: No

Reviewer #3: No

[NOTE: If reviewer comments were submitted as an attachment file, they will be attached to this email and accessible via the submission site. Please log into your account, locate the manuscript record, and check for the action link "View Attachments". If this link does not appear, there are no attachment files.]

**Figure resubmission:**

While revising your submission, we strongly recommend that you use PLOS’s NAAS tool (https://ngplosjournals.pagemajik.ai/artanalysis) to test your figure files. NAAS can convert your figure files to the TIFF file type and meet basic requirements (such as print size, resolution), or provide you with a report on issues that do not meet our requirements and that NAAS cannot fix.

After uploading your figures to PLOS’s NAAS tool - https://ngplosjournals.pagemajik.ai/artanalysis, NAAS will process the files provided and display the results in the "Uploaded Files" section of the page as the processing is complete. If the uploaded figures meet our requirements (or NAAS is able to fix the files to meet our requirements), the figure will be marked as "fixed" above. If NAAS is unable to fix the files, a red "failed" label will appear above. When NAAS has confirmed that the figure files meet our requirements, please download the file via the download option, and include these NAAS processed figure files when submitting your revised manuscript.

**Reproducibility:**

To enhance the reproducibility of your results, we recommend that authors of applicable studies deposit laboratory protocols in protocols.io, where a protocol can be assigned its own identifier (DOI) such that it can be cited independently in the future. Additionally, PLOS ONE offers an option to publish peer-reviewed clinical study protocols. Read more information on sharing protocols at https://plos.org/protocols?utm_medium=editorial-email&utm_source=authorletters&utm_campaign=protocols

# Author comment: Author response to Decision Letter 1

**DOI:** 10.1371/journal.pntd.0014857.r002

29 Jul 2026

Attachment Submitted filename: Authors response to editor and reviewers.docx

# Peer review: Decision Letter 1

**DOI:** 10.1371/journal.pntd.0014857.r003

**Authors:** Peter Fischer

22 Oct 2026

PNTD-D-26-00780R1Tracing Strongyloides stercoralis Transmission and Risk Factors from Schoolchildren to Household in Northwest EthiopiaPLOS Neglected Tropical Diseases Dear Dr. Hailu, Thank you for submitting your manuscript to PLOS Neglected Tropical Diseases. After careful consideration, we feel that it has merit but does not fully meet PLOS Neglected Tropical Diseases's publication criteria as it currently stands. Therefore, we invite you to submit a revised version of the manuscript that addresses the points raised during the review process. Please submit your revised manuscript by Dec 21 2026 11:59PM. If you will need more time than this to complete your revisions, please reply to this message or contact the journal office at plosntds@plos.org. When you're ready to submit your revision, log on to https://www.editorialmanager.com/pntd/ and select the 'Submissions Needing Revision' folder to locate your manuscript file. Please include the following items when submitting your revised manuscript:* A letter that responds to each point raised by the editor and reviewer(s). You should upload this letter as a separate file labeled 'Response to Reviewers'. This file does not need to include responses to any formatting updates and technical items listed in the 'Journal Requirements' section below.* A marked-up copy of your manuscript that highlights changes made to the original version. You should upload this as a separate file labeled 'Revised Manuscript with Track Changes'.* An unmarked version of your revised paper without tracked changes. You should upload this as a separate file labeled 'Manuscript'. If you would like to make changes to your financial disclosure, competing interests statement, or data availability statement, please make these updates within the submission form at the time of resubmission. Guidelines for resubmitting your figure files are available below the reviewer comments at the end of this letter. As the corresponding author, your ORCID iD is verified in the submission system and will appear in the published article. PLOS supports the use of ORCID, and we encourage all coauthors to register for an ORCID iD and use it as well. Please encourage your coauthors to verify their ORCID iD within the submission system before final acceptance, as unverified ORCID iDs will not appear in the published article. Only the individual author can complete the verification step; PLOS staff cannot verify ORCID iDs on behalf of authors.We look forward to receiving your revised manuscript. Kind regards, Peter U FischerSection EditorPLOS Neglected Tropical Diseases Peter FischerSection EditorPLOS Neglected Tropical Diseases

Shaden Kamhawi

co-Editor-in-Chief

PLOS Neglected Tropical Diseases

orcid.org/0000-0003-4304-636XX

Paul Brindley

co-Editor-in-Chief

PLOS Neglected Tropical Diseases

orcid.org/0000-0003-1765-0002

**Reviewers' comments:**  Reviewer's Responses to Questions

**Key Review Criteria Required for Acceptance?**

As you describe the new analyses required for acceptance, please consider the following:

**Methods**

-Are the objectives of the study clearly articulated with a clear testable hypothesis stated?

-Is the study design appropriate to address the stated objectives?

-Is the population clearly described and appropriate for the hypothesis being tested?

-Is the sample size sufficient to ensure adequate power to address the hypothesis being tested?

-Were correct statistical analysis used to support conclusions?

-Are there concerns about ethical or regulatory requirements being met?

Reviewer #1: (No Response)

Reviewer #3: Yes

**********

**Results**

-Does the analysis presented match the analysis plan?

-Are the results clearly and completely presented?

-Are the figures (Tables, Images) of sufficient quality for clarity?

Reviewer #1: (No Response)

Reviewer #3: Yes

**********

**Conclusions**

-Are the conclusions supported by the data presented?

-Are the limitations of analysis clearly described?

-Do the authors discuss how these data can be helpful to advance our understanding of the topic under study?

-Is public health relevance addressed?

Reviewer #1: The authors have made substantial revisions to the manuscript and have addressed most of the concerns raised during the previous review. However, a few issues still require clarification or correction before the manuscript can be considered for acceptance.

1. The major concern regarding the interpretation of household transmission has been partially addressed. The authors now appropriately acknowledge that longitudinal and molecular epidemiological studies are required to determine transmission pathways. However, statements such as “ongoing household transmission” and “sustained intra-household transmission” remain stronger than supported by the cross-sectional study design. The current data support household or familial clustering of infection but cannot establish direct transmission or its directionality. Please ensure that this distinction is consistently reflected throughout the Abstract, Authors’ Summary, and Conclusion.

2. The authors have appropriately acknowledged the possibility of participation bias because only 23 of the 62 eligible households participated in household screening. However, it remains unclear whether the characteristics of participating and non-participating households could be compared. If such data are unavailable, this should be explicitly stated, and the potential impact of participation bias on the estimated household prevalence and its generalizability should be clearly acknowledged.

Reviewer #3: Yes

**********

**Editorial and Data Presentation Modifications?**

Use this section for editorial suggestions as well as relatively minor modifications of existing data that would enhance clarity. If the only modifications needed are minor and/or editorial, you may wish to recommend “Minor Revision” or “Accept”.

Reviewer #1: (No Response)

Reviewer #3: (No Response)

**********

**Summary and General Comments**

Use this section to provide overall comments, discuss strengths/weaknesses of the study, novelty, significance, general execution and scholarship. You may also include additional comments for the author, including concerns about dual publication, research ethics, or publication ethics. If requesting major revision, please articulate the new experiments that are needed.

Reviewer #1: The authors have made substantial revisions to the manuscript and have addressed most of the concerns raised during the previous review. However, a few issues still require clarification or correction before the manuscript can be considered for acceptance.

1. The major concern regarding the interpretation of household transmission has been partially addressed. The authors now appropriately acknowledge that longitudinal and molecular epidemiological studies are required to determine transmission pathways. However, statements such as “ongoing household transmission” and “sustained intra-household transmission” remain stronger than supported by the cross-sectional study design. The current data support household or familial clustering of infection but cannot establish direct transmission or its directionality. Please ensure that this distinction is consistently reflected throughout the Abstract, Authors’ Summary, and Conclusion.

2. The authors have appropriately acknowledged the possibility of participation bias because only 23 of the 62 eligible households participated in household screening. However, it remains unclear whether the characteristics of participating and non-participating households could be compared. If such data are unavailable, this should be explicitly stated, and the potential impact of participation bias on the estimated household prevalence and its generalizability should be clearly acknowledged.

Reviewer #3: The authors have addressed my all previous comments.

**********

PLOS authors have the option to publish the peer review history of their article (what does this mean?). If published, this will include your full peer review and any attached files.

If you choose “no”, your identity will remain anonymous but your review may still be made public.

**Do you want your identity to be public for this peer review?**  For information about this choice, including consent withdrawal, please see our Privacy Policy.

Reviewer #1: No

Reviewer #3: No

[NOTE: If reviewer comments were submitted as an attachment file, they will be attached to this email and accessible via the submission site. Please log into your account, locate the manuscript record, and check for the action link "View Attachments". If this link does not appear, there are no attachment files.] **Figure resubmission:**  While revising your submission, we strongly recommend that you use PLOS’s NAAS tool (https://ngplosjournals.pagemajik.ai/artanalysis) to test your figure files. NAAS can convert your figure files to the TIFF file type and meet basic requirements (such as print size, resolution), or provide you with a report on issues that do not meet our requirements and that NAAS cannot fix.

After uploading your figures to PLOS’s NAAS tool - https://ngplosjournals.pagemajik.ai/artanalysis, NAAS will process the files provided and display the results in the "Uploaded Files" section of the page as the processing is complete. If the uploaded figures meet our requirements (or NAAS is able to fix the files to meet our requirements), the figure will be marked as "fixed" above. If NAAS is unable to fix the files, a red "failed" label will appear above. When NAAS has confirmed that the figure files meet our requirements, please download the file via the download option, and include these NAAS processed figure files when submitting your revised manuscript. **Reproducibility:**  To enhance the reproducibility of your results, we recommend that authors of applicable studies deposit laboratory protocols in protocols.io, where a protocol can be assigned its own identifier (DOI) such that it can be cited independently in the future. Additionally, PLOS ONE offers an option to publish peer-reviewed clinical study protocols. Read more information on sharing protocols at https://plos.org/protocols?utm_medium=editorial-email&utm_source=authorletters&utm_campaign=protocols

# Author comment: Author response to Decision Letter 2

**DOI:** 10.1371/journal.pntd.0014857.r004

23 Oct 2026

Attachment Submitted filename: Response to Reviewers.docx

# Editor report: Decision Letter 2

**DOI:** 10.1371/journal.pntd.0014857.r005

**Authors:** Peter Fischer

26 Oct 2026

Dear Dr Hailu,

We are pleased to inform you that your manuscript 'Tracing Strongyloides stercoralis Transmission and Risk Factors from Schoolchildren to Household in Northwest Ethiopia' has been provisionally accepted for publication in PLOS Neglected Tropical Diseases.

Before your manuscript can be formally accepted you will need to complete some formatting changes, which you will receive in a follow up email. A member of our team will be in touch with a set of requests.

Please note that your manuscript will not be scheduled for publication until you have made the required changes, so a swift response is appreciated.

IMPORTANT: The editorial review process is now complete. PLOS will only permit corrections to spelling, formatting or significant scientific errors from this point onwards. Requests for major changes, or any which affect the scientific understanding of your work, will cause delays to the publication date of your manuscript.

Should you, your institution's press office or the journal office choose to press release your paper, you will automatically be opted out of early publication. We ask that you notify us now if you or your institution is planning to press release the article. All press must be co-ordinated with PLOS.

Thank you again for supporting Open Access publishing; we are looking forward to publishing your work in PLOS Neglected Tropical Diseases.

Best regards,

Peter U Fischer

Section Editor

PLOS Neglected Tropical Diseases

Peter Fischer

Section Editor

PLOS Neglected Tropical Diseases

Shaden Kamhawi

co-Editor-in-Chief

PLOS Neglected Tropical Diseases

orcid.org/0000-0003-4304-636XX

Paul Brindley

co-Editor-in-Chief

PLOS Neglected Tropical Diseases

orcid.org/0000-0003-1765-0002

***********************************************************

# Editor report: Acceptance letter

**DOI:** 10.1371/journal.pntd.0014857.r006

**Authors:** Peter Fischer

Dear Dr Hailu,

We are delighted to inform you that your manuscript, "Tracing Strongyloides stercoralis Transmission and Risk Factors from Schoolchildren to Household in Northwest Ethiopia," has been formally accepted for publication in PLOS Neglected Tropical Diseases.

We have now passed your article onto the PLOS Production Department who will complete the rest of the publication process. All authors will receive a confirmation email upon publication.

The corresponding author will soon be receiving a typeset proof for review, to ensure errors have not been introduced during production. Please review the PDF proof of your manuscript carefully, as this is the last chance to correct any scientific or type-setting errors. Please note that major changes, or those which affect the scientific understanding of the work, will likely cause delays to the publication date of your manuscript. Note: Proofs for Front Matter articles (Editorial, Viewpoint, Symposium, Review, etc...) are generated on a different schedule and may not be made available as quickly.

Soon after your final files are uploaded, the early version of your manuscript will be published online unless you opted out of this process. The date of the early version will be your article's publication date. The final article will be published to the same URL, and all versions of the paper will be accessible to readers.

For Research Articles, you will receive an invoice from PLOS for your publication fee after your manuscript has reached the completed accept phase. If you receive an email requesting payment before acceptance or for any other service, this may be a phishing scheme. Learn how to identify phishing emails and protect your accounts at https://explore.plos.org/phishing.

Thank you again for supporting open-access publishing; we are looking forward to publishing your work in PLOS Neglected Tropical Diseases.

Best regards,

Shaden Kamhawi

co-Editor-in-Chief

PLOS Neglected Tropical Diseases

Paul Brindley

co-Editor-in-Chief

PLOS Neglected Tropical Diseases
